# White Light Emission from Fluorescent SiC with Porous Surface

**DOI:** 10.1038/s41598-017-10771-7

**Published:** 2017-08-29

**Authors:** Weifang Lu, Yiyu Ou, Elisabetta Maria Fiordaliso, Yoshimi Iwasa, Valdas Jokubavicius, Mikael Syväjärvi, Satoshi Kamiyama, Paul Michael Petersen, Haiyan Ou

**Affiliations:** 1 Department of Photonics Engineering, Technical University of Denmark, DK-2800 Kgs, Lyngby Denmark; 2 Center for Electron Nanoscopy, Technical University of Denmark, Fysikvej 307, DK-2800 Kgs, Lyngby Denmark; 3 Department of Materials Science and Engineering, Meijo University,1-501 Shiogamaguchi, Tenpaku-ku, Nagoya 468-8502 Japan; 4 Department of Physics, Chemistry and Biology, Linköping University, SE-58183 Linköping, Sweden

## Abstract

We report for the first time a NUV light to white light conversion in a N-B co-doped 6H-SiC (fluorescent SiC) layer containing a hybrid structure. The surface of fluorescent SiC sample contains porous structures fabricated by anodic oxidation method. After passivation by 20 nm thick Al_2_O_3_, the photoluminescence intensity from the porous layer was significant enhanced by a factor of more than 12. Using a porous layer of moderate thickness (~10 µm), high-quality white light emission was realized by combining the independent emissions of blue-green emission from the porous layer and yellow emission from the bulk fluorescent SiC layer. A high color rendering index of 81.1 has been achieved. Photoluminescence spectra in porous layers fabricated in both commercial n-type and lab grown N-B co-doped 6H-SiC show two emission peaks centered approximately at 460 nm and 530 nm. Such blue-green emission phenomenon can be attributed to neutral oxygen vacancies and interface C-related surface defects generated dring anodic oxidation process. Porous fluorescent SiC can offer a great flexibility in color rendering by changing the thickness of porous layer and bulk fluorescent layer. Such a novel approach opens a new perspective for the development of high performance and rare-earth element free white light emitting materials.

## Introduction

White light-emitting materials have been intensively investigated because of their potential in a wide range of applications in illumination and display systems^1–5^. Currently, there are mainly three approaches to generate white light by using light-emitting diodes (LEDs): mixing red, green, and blue (RGB) LEDs^6^; blue LEDs covered with yellow-emitting phosphors^4, 7^; using a near ultraviolet (NUV) LED to stimulate RGB phosphors^8^. The RGB-LEDs based white light sources provide possibility to vary the color-rendering index (CRI) and luminous efficacy^5, 6^. However, it is a key challenge to maintain the desired stability in terms of CRI and correlated color temperature (CCT), which always varies with ambient temperature and time^9^. Nowadays, most of commercial white light LEDs use a blue LED chip covered by a yellowish phosphor layer such as cerium-doped yttrium aluminum garnets (YAG: Ce)^4, 7, 10–14^. Unfortunately, the degradation of the blue LED chips or phosphors at different rate can lead to poor CRI and furthermore low stability of CCT. In contrast, white light sources fabricated by using NUV-LED chips and RGB phosphors might overcome those disadvantages, due to the invisibility of the NUV light^8, 10^. But it has been reported that red nitride phosphors doped with rare-earth elements absorb not only the NUV light as desired but also the blue-green light generated from the blue and green phosphors but lost as heat, which can decrease the total luminous efficiency^10^. Accordingly, the red phosphors for NUV-LEDs based white light sources need further development.

Fluorescent 6H-SiC (band gap = 3.0 eV) co-doped with donor and acceptor is a promising candidate as a phosphor material in NUV-LEDs based white light sources without using rare-earth elements^15^. Silicon carbide is a well-established material for epitaxial growth of nitride layers for NUV-LEDs. Also, due to high thermal conductivity it is an excellent semiconductor material for high power applications. Due to the large bandgap of 6H-SiC material, nitrogen-boron (N-B) and nitrogen-aluminum (N-Al) in 6H-SiC can provide broadband donor-acceptor pairs (DAP) emission near the yellow and blue spectral region, respectively^16, 17^. By combining the broadband yellow and blue light, white light with a high CRI can be achieved. However, currently, there are still several limitations to realize white light emission by combining N-B and N-Al co-doped SiC epilayers. One of them is the lower efficiency of N-Al DAP emission compared to N-B DAP emission due to the significant thermal ionization of Al acceptor states^18^. Therefore, the N-Al DAP emission at 460 nm has so far only been observed at extremely low temperature near 4 K since the polarization interaction of Al acceptors is dominant at high temperature^19–21^.

Herein, we proposed an innovative method to achieve white light emission by converting NUV light in a hybrid structure composed of N-B co-doped 6H-SiC and porous layer formed on top of it. The porous SiC layer can emit broadband blue-green light which is attributed to the surface defects related emission^22–24^ and the luminescence intensity is rather stable after passivation by atomic layer deposited (ALD) dielectric films^25, 26^. By combining the blue-green emission from the porous layer and the yellow emission from the fluorescent SiC substrate, the emitting spectrum can cover the whole visible range. We believe our detailed studies could provide understanding the luminescence mechanisms in porous SiC to realize white light emitting with high CRI and high luminescence efficiency.

## Results and Discussion

### Sample Preparation and Morphology Characterization of Porous SiC

The fluorescent 6H-SiC samples were grown by the fast sublimation growth process (FSGP) at 1900 °C in nitrogen ambient (10^−2^ mbar)^27^. The doping concentration measured by secondary ion mass spectroscopy is 1.3 × 10^18^ cm^−3^ and 0.9 × 10^18^ cm^−3^ for N and B, respectively. For comparison, commercially available 6H-SiC substrate (n-type, background doping level of N) supplied by SiCrystal AG (Germany) was also used in this study for porous structure fabrication. The commercial SiC substrate (SiC-Sub) with a thickness of 250 µm was Si-face polished. To achieve ohmic contacts on the backside of SiC samples, layered nickel (100 nm)/titanium (10 nm)/gold (200 nm) films were prepared by sputtering or e-beam evaporation processes. An anodic etching process was executed to form the porous layers on the SiC substrate as described in our previous publication^26^. The samples were immersed in a 5% HF solution and exposed under the UV irradiation of 365 nm by using mercury lamp. Four porous samples with different anodic etching durations were prepared for light emission investigations, which are referred as samples a, b, c, and d, as listed in Table 1. The anodic etching duration for SiC-Sub sample a, fluorescent samples b, c and d was 960 min, 960 min, 200 min, and 500 min, respectively. All the fluorescent samples were immersed in HF solution with 0.015 mol/L of K_2_S_2_O_8_ during etching. The thickness of the porous layer measured by scanning electron microscope (SEM) for samples a, b, c and d is 13 µm, 21.4 µm, 4.6 µm and 10 µm, respectively. Since fluorescent SiC samples are doped with N and B, the anodic etching rate (~20 nm/min) is higher than that for the SiC-Sub sample (~13 nm/min).

**Table 1:** Anodic Oxidation Conditions for Porous Samples a, b, c, and d.

Samples	Sub. types	Dopants	Anodic etching duration	Oxidant	Porous thickness
a	Commercial 6H-SiC substrate (SiC-Sub)	N(background doping level)	960 min	0 mol/L	13 µm
b	Fluorescent 6H-SiC	B, N	960 min	0.015 mol/L	21.4 µm
c	Fluorescent 6H-SiC	B, N	200 min	0.015 mol/L	4.6 µm
d	Fluorescent 6H-SiC	B, N	500 min	0.015 mol/L	10 µm

**Figure 1:**
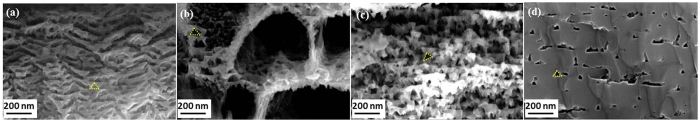
Cross-sectional SEM images of porous samples after anodic etching: (**a**) and (**b**) show the top and bottom layer of the sample a respectively, while (**c**) and (**d**) are for the sample d with 0.015 mol/L K_2_S_2_O_8_ (pores are marked by yellow triangles).

SEM images were acquired from a cross section of the samples a and d, to visualize the porous structure along the depth of each layer, as shown in Fig. 1. Images were acquired at the top and the bottom of the porous layer in the sample a (Fig. 1(a) and (b)). The SEM images show that the porosity is non-uniform along the depth and a similar result is observed for the fluorescent sample without oxidant (not shown). In order to improve the porosity uniformity, potassium persulfate (K_2_S_2_O_8_) was introduced during the fabrication of the porous structure with a concentration of 0.015 mol/L. The SEM images of the top and bottom layer of fluorescent sample d with K_2_S_2_O_8_ are shown in Fig. 1(c) and (d), respectively. The uniformity of porosity near the bottom area in sample d is much better than that in sample a.

**Figure 2:**
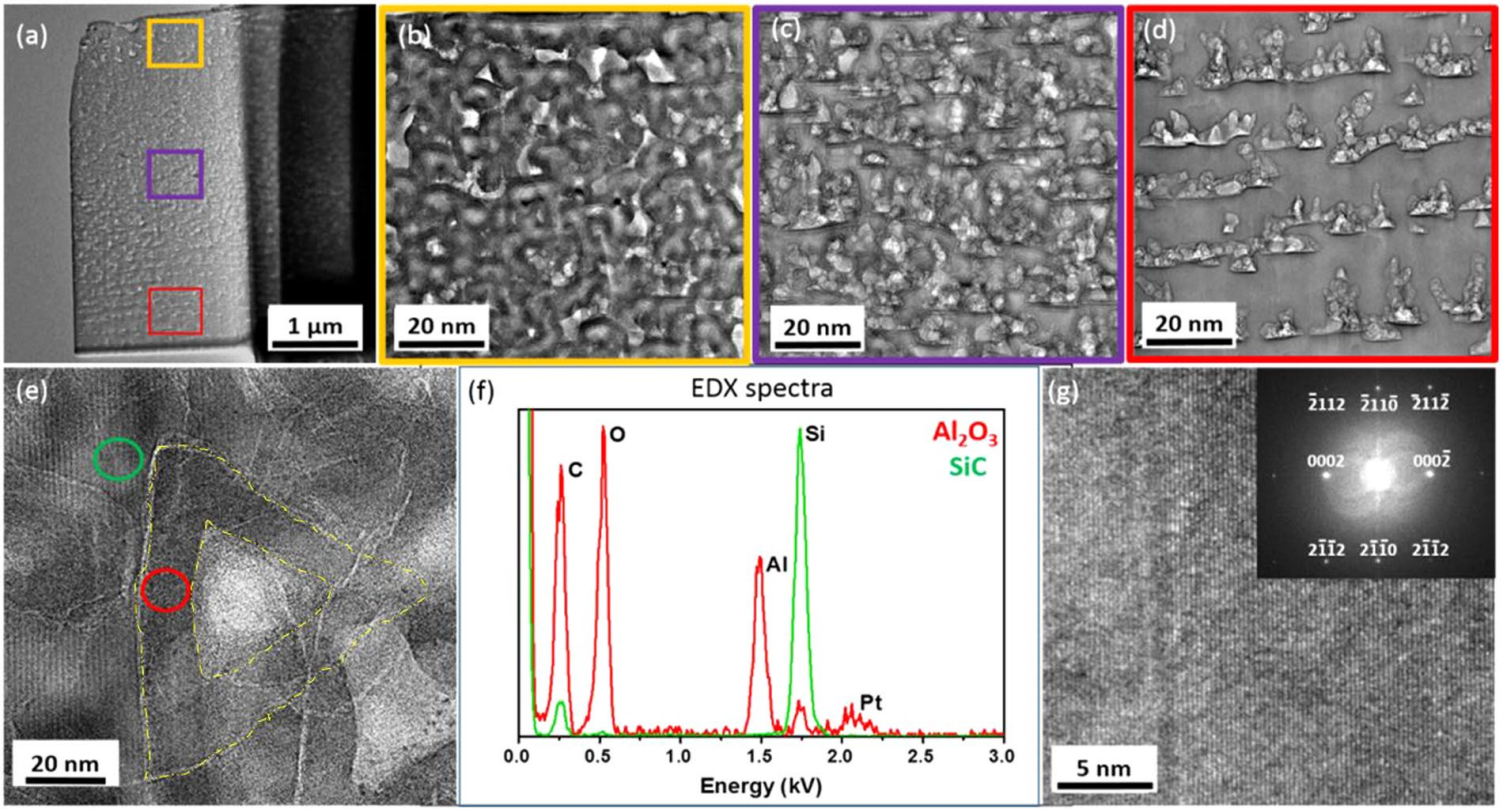
TEM images of porous 6H-SiC sample d after ALD passivation with 20 nm Al_2_O_3_. (**a**) The overview of the fibbed lamella. (**b**–**d**): top, middle and bottom layer of the sample. (**e**): an area around a pore, marked by dash yellow line. (**f**) EDX spectra acquired from the green and red circles in (**e**). (**g**): high resolution image of 6H-SiC area with corresponding FFT as an inset.

All samples were passivated by 20 nm ALD Al_2_O_3_ in order to decrease the non-radiative recombination due to surface defects. Sample d was characterized by transmission electron microscopy (TEM), after preparing a thin lamella by focused ion beam (FIB). Figure 2(a) shows a low magnification TEM image of the fibbed lamella, whereas (b), (c) and (d) are images acquired at higher magnification from the top, the middle and the bottom area of the layer marked by yellow, purple and red squares respectively for the investigation of porous structure change as a function of depth. It can be seen that the porosity decreases from the top area to the bottom area of porous layer. The pores in top area are almost full-filled with Al_2_O_3_ films (the scattered white areas are not full-filled pores), while the pores in deep area are covered with thinner layer of Al_2_O_3_. Figure 2(e) shows a TEM image acquired around a pore in the middle of the layer. A dark layer of ~20 nm is observed around the pore and this is consistent with the expected Al_2_O_3_ thickness. Energy dispersive X-ray (EDX) spectra were acquired at two different locations in order to obtain information on the chemical composition. EDX spectra acquired from the green and red circles in Fig. 2(e) are presented in Fig. 2(f) which indicates that the layer around the pore (area between dashed yellow lines) contains mostly Al_2_O_3_, while SiC is detected in between the pores. The fluorescent 6H-SiC has a crystalline structure as shown in the high resolution TEM image reported in Fig. 2(g). From the corresponding fast Fourier transform (FFT) image shown as an inset of Fig. 2(g), the zone axis [$1{\rm{1}}\overline{{\rm{2}}}{\rm{0}}$] of 6H-SiC is identified. It can be seen that the fluorescent SiC exhibits the hexagonal crystal structure.

### PL Enhancement in Porous SiC Passivated by ALD Al_2_O_3_ Films

After porous structure fabrication, a tremendous density of surface defects were induced into porous SiC layer, which create non-radiative recombination centers (dangling bonds) and decrease the PL intensity. Remarkably, the Al_2_O_3_ films synthesized by ALD demonstrated significant surface passivation effect on porous SiC. Based on our previous effective optimization for surface passivation on porous SiC, a 20 nm thick Al_2_O_3_ layer was used to passivate the surface of porous SiC^26^. The Al_2_O_3_ film was deposited at 160 °C with purge time of 20 s, followed by an annealing for 5 min at 350 °C.

**Figure 3:**
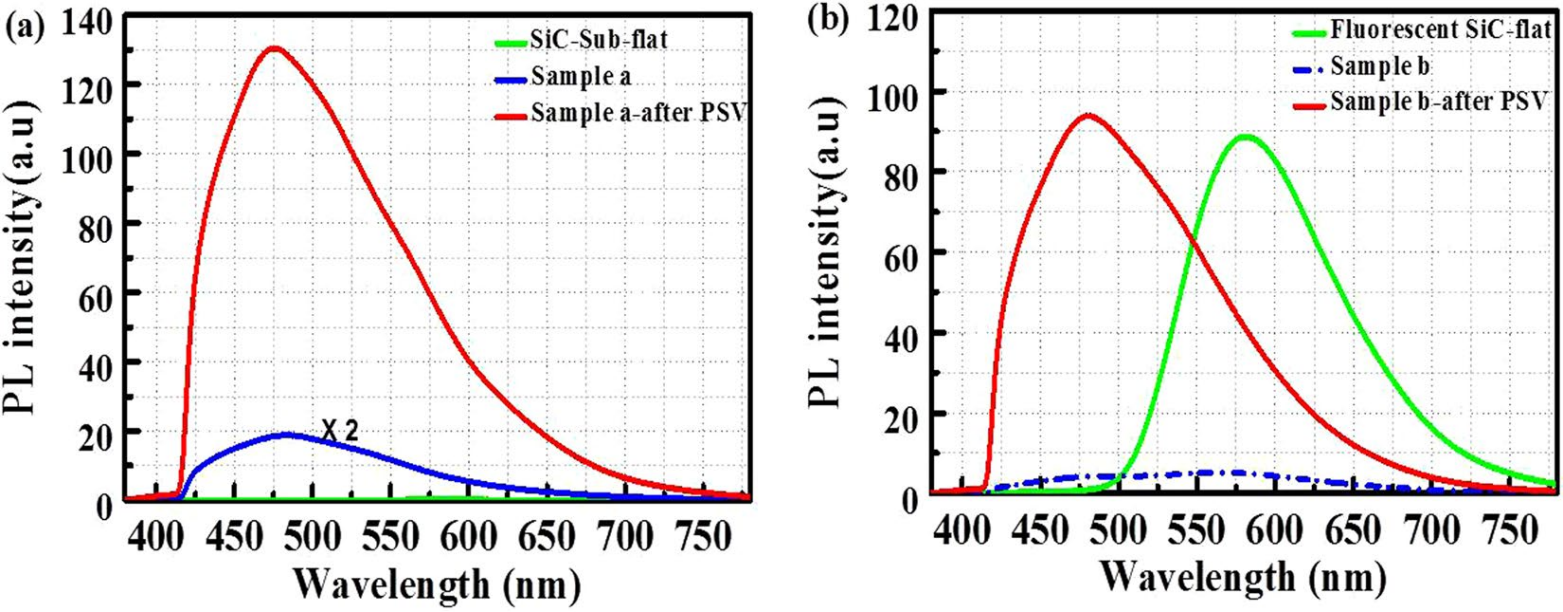
PL spectra of porous samples a and b before and after passivation (PSV) by 20 nm thick Al_2_O_3_. (**a**) Porous sample a on SiC-sub, the PL intensity has been improved by a factor of 12.9 after passivation. (**b**) Porous sample b on fluorescent SiC, the PL intensity enhancement reaches 19.8. The PL spectra of untreated flat substrates (labelled as “SiC-Sub-flat” and “Fluorescent SiC-flat”) are also shown for comparison.

The passivation effect is characterized by PL measurements using a 375 nm laser as excitation source coupled into an optical microscope. The collected emission signal was filtered by a long-pass filter with a cutoff wavelength at 420 nm. The PL spectra of porous samples a and b before and after passivation are shown in Fig. 3. After passivation, the PL intensity of porous samples a and b increased by a factor of 12.9 and 19.8, respectively. It is interesting to note that the emission peaks in the SiC-Sub and fluorescent SiC are located at the same position around 475 nm. Without DAP emission, the PL intensity in flat SiC-Sub is extremely weak, as shown in Fig. 3(a). Nevertheless, after passivation of Al_2_O_3_ by ALD, the PL intensity of porous sample a is comparable to that in sample b. It can be concluded that the emission from the porous layer of SiC is irrelevant to N and B dopants. In Fig. 3(b), the emission in sample b (the porous layer is 21.4 µm thick) dramatically quenched after porous structure fabrication due to non-radiative recombination. Since the penetration depth of 375 nm light in SiC is about 15 µm, the DAP emission in the bulk layer almost can’t be excited due to the absorption by the thick porous layer on top. In addition, the full width at half maximum (FWHM) of passivated sample a and b is 137 nm and 145 nm, respectively. From such wide FWHM in porous SiC, one can be inspired to speculate that there might be several photoluminescence mechanisms in porous SiC. The flat SiC substrates were also passivated by 20 nm thick Al_2_O_3_ with the same conditions. However, no PL change due to Al_2_O_3_ has been observed, which excludes the luminescence from Al_2_O_3_ films. The main contribution for ALD Al_2_O_3_ is the passivation effect by eliminating the non-radiative recombination centers (such as dangling bonds) on the porous surface. These results verify the passivation effect of ALD Al_2_O_3_ films on porous fluorescent SiC for color-conversion.

### White Light Emission from Porous Fluorescent SiC

**Figure 4:**
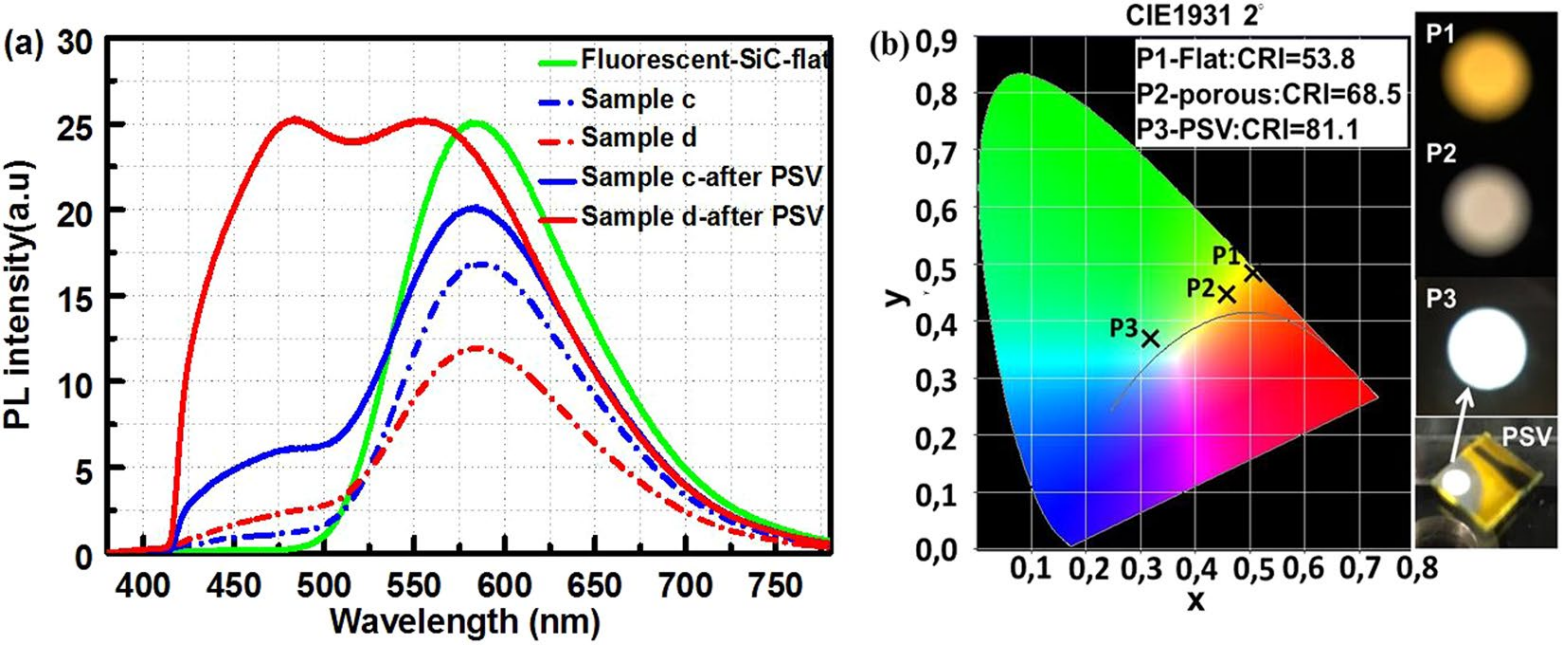
(**a**) PL spectra of porous fluorescent 6H-SiC samples c and d before and after passivation by 20 nm thick Al_2_O_3_: the thickness of the porous layer is 4.6 µm and 10 µm respectively. (**b**) Chromaticity diagram showing the color of the light emitted from fluorescent flat (P1), porous sample d before (P2) and after passivation (P3), the corresponding CRI is 53.8, 68.5, and 81.1, respectively. On the right of Fig. 4(b), camera photos corresponding to P1, P2, and P3 are shown.

To obtain white light emission with a good quality from porous fluorescent SiC, the porous thickness was tuned by adjusting the anodic oxidation duration. In an initial attempt, sample c and d were fabricated with a porous thickness of 4.6 µm and 10 µm on the same fluorescent SiC substrate (50 µm thick), respectively. The PL spectra of the as-grown and porous samples before and after passivation are shown in Fig. 4(a). The spectra were collected by using a 375 nm laser as excitation source at room temperature. There are two distinct peaks located around 475 nm and 580 nm which correspond to the emission from the porous and fluorescent layers. After ALD passivation by 20 nm thick Al_2_O_3_, the porous emission in these porous samples is significantly improved, especially for sample d. The FWHM of PL spectrum from sample d is increased from 108.4 nm in as-grown substrate to 205.9 nm. Notably, the PL spectrum of porous sample c has rather weak emission, compared with the DAP emission peak.

The corresponding CRI for sample d with porous surface is 68.5, while that in the original flat substrate is 53.8. As expected, the CRI in sample d is increased to 81.1 after ALD passivation. In addition, the correlated color temperature (CCT) of sample d with passivation is 6034 K which corresponds to a “cool” white light. In this way, the emission color in fluorescent SiC was tuned from yellow to pure white by fabricating an optimized thickness of porous layer and ALD Al_2_O_3_ passivation layer. The corresponding luminescence points in flat substrate, sample d before and after passivation is shown in the Commission International de I’Eclairage (CIE, 1931) chromaticity diagram in Fig. 4(b). The warm yellow to bright white light luminescence emitted from porous fluorescent is strong enough to be observed by the naked eyes under laser excitation, as shown in the inset camera photos in Fig. 4(b). It is worth mentioning that the proportional distribution of the two emissions can be tuned by adjusting the thickness of the fluorescent bulk layer and the surface porous layer. The tunable luminescent properties of porous fluorescent SiC offer a perspective to obtain white light emission with rather high CRI.

### Raman Spectroscopy of Porous SiC

**Figure 5:**
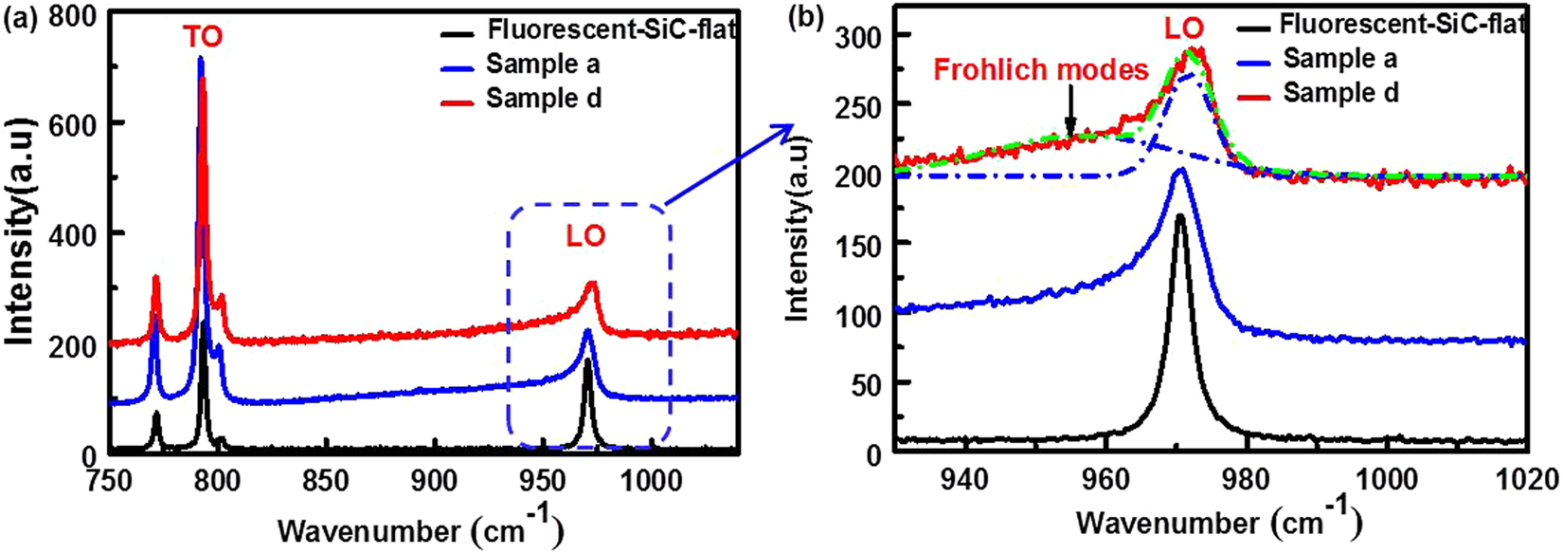
(**a**) Raman spectra of flat 6H-SiC, porous sample a and d. Two prominent peaks at around 793 cm^−1^ and 970 cm^−1^ are present in the spectra. (**b**) The Raman spectra only show the LO mode around 970 cm^−1^. The spectrum of sample d was fitted with a Gaussian bulk LO mode and surface Fröhlich modes.

The origin of the emission peaks from porous SiC could mainly be from either quantum confinement effect or surface states/defects^24, 28^. Since the Bohr radius of 6H-SiC is only 0.7 nm^28^ and the pore size in this work is about 40 nm, the quantum confinement effect in SiC-Sub or fluorescent 6H-SiC can be ruled out as an important role in the porous emission. In addition, the emission peaks in SiC-Sub or fluorescent SiC porous samples are located at the same wavelength near 480 nm, and the emission intensity is also similar. To further understand the emission mechanism in porous SiC, the Raman spectra recorded in the range 750–1040 cm^−1^ were analyzed. As shown in Fig. 5(a), the spectra in flat fluorescent sample, sample a and d exhibit intense peaks at around 793 cm^−1^ and 970 cm^−1^, corresponding to the transverse optical phonons (TO) and longitudinal optical phonon (LO) frequencies.

The Raman spectra were reported to discern the quantum confinement effect by LO mode^24^. The LO mode under quantum confinement effect should exhibit a slight broadening accompanied by shift to lower frequency^24^. However, as seen in Fig. 5(b), porous samples a and d show asymmetric broadening and slightly shift to higher frequency, compared with the flat substrate. The asymmetry of the LO mode is a measure of various properties of the material, such as surface defect states, amorphous features and size of nanocrystals. An explanation for the asymmetric broadening of Raman LO mode in porous SiC is the emerging of Fröhlich modes^29, 30^. The origin of Fröhlich mode is ascribed to the defects on the surface of pores, because the enormous amount of surface defects leads to large exciton polarization thus coupled with LO vibration. For instance, the Raman spectrum of sample d was fitted with two components including a Gaussian LO phonon mode of bulk SiC (971.3 cm^−1^) and a peak due to the surface defects related Fröhlich modes (956.7 cm^−1^). It is thus verified that the surface defects is the dominant reason giving rise to asymmetric broadening of LO mode in porous SiC samples. Accordingly, it is reasonable to assume the blue-green emission stems from surface defects^31^.

### Photoluminescence Mechanisms in Porous SiC

**Figure 6:**
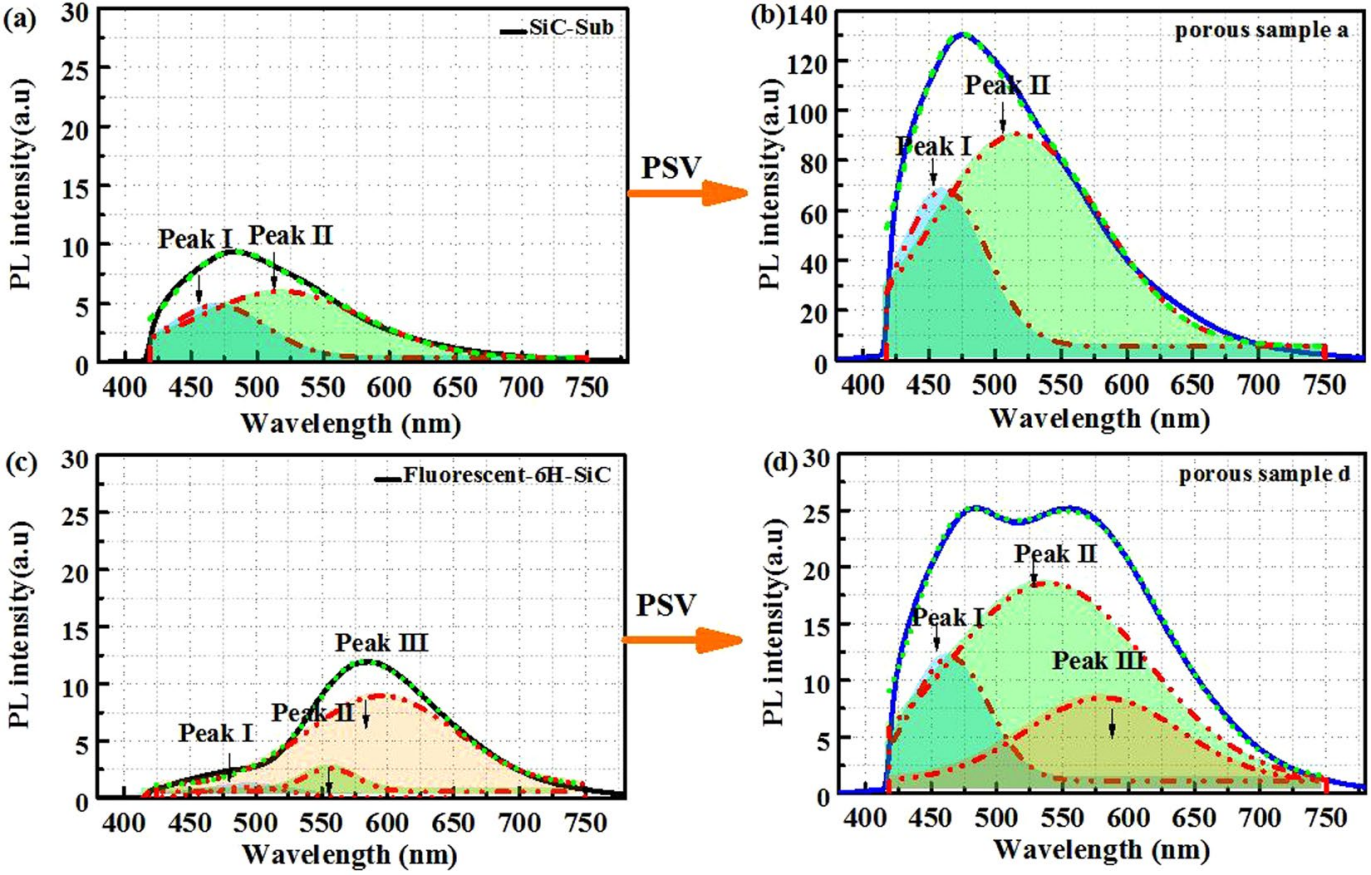
PL spectra with Gaussian fitting of porous samples a (SiC-Sub): (**a**) before and (**b**) after passivation by 20 nm Al_2_O_3_, the curves were fitted with two emission peaks centered at 460 nm (peak I) and 520 nm (peak II); for porous sample d (fluorescent 6H-SiC) (**c**) before and (**d**) after the passivation, the DAP recombination band located near 580 nm (peak III) was added into fitting.

In order to assess the contribution from different radiative recombination channels, the PL spectra of porous sample a and d were deconvoluted into Gaussian-like peaks^32^, as shown in Fig. 6. In porous sample a, the PL spectra are fitted by two Gaussian peaks: peak I centered at approximately 460 nm and peak II centered at roughly 520 nm. In porous fluorescent sample d, one more component was added into the fitting due to DAP emission located at around 580 nm (peak III). The peak position of peak II is slightly red-shifted in fluorescent porous sample. It can be seen that the intensity of the peak I and peak II in the porous layer was significantly enhanced after surface passivation. Meanwhile, the DAP emission peak in porous sample d almost remained unchanged. Based on the aforementioned results, the broadband luminescence in porous SiC are attributed to surface defects^33, 34^, such as neutral oxygen vacancies in surface oxide layer, interface terminations (dangling bonds), special rough interface and structure defects containing tremendous C-related defects.

Herein, the PL emission peak I of the porous SiC can be attributed to the oxygen vacancy in the porous structures. The existence of oxygen vacancies, emitting in the blue light range of 410–470 nm, is a common phenomenon in semiconductor materials^28, 35–37^. It has been reported that the luminescence peaks at 460 nm (2.7 eV) was derived from the triplet-to-ground transition of a neutral oxygen vacancy defect (O_3_≡Si-Si≡O_3_)^36^. During anodic oxidation process, a large number of oxidized surface groups were introduced into porous SiC structures because of the applied strong acids reaction between SiC and OH^-^. Various oxygen-containing surface defects contribute to the luminescence, leading to a relatively broad emission peak at room temperature. The broad blue-green emission peak II at ~530 nm (2.3 eV) is due to non-bridging oxygen hole centers (O_3_≡Si-O^●^)^38^ and C-related defect centers located near the interface^34, 39^. It is known that the SiC reacting with HF can produce a C-enriched phase at the surface and provide a high density of C-related surface defects^40^. The higher defect density will lead to the enhancement of porous emission. Since interface termination defect can also provide non-radiative recombination channels, the porous SiC samples need to be passivated by ALD Al_2_O_3_ films^25^. During anodic oxidation process, the oxygen atoms were incorporated into Si-C bonds at the interface. When the oxygen atoms diffuse and react with SiC during anodic oxidation etching, dangling bond defects are generated due to the incomplete formation of Si-C bonds^41^. Therefore, the ALD Al_2_O_3_ films can effectively passivate the dangling bond defects by hydrogen saturation^26^.

**Figure 7:**
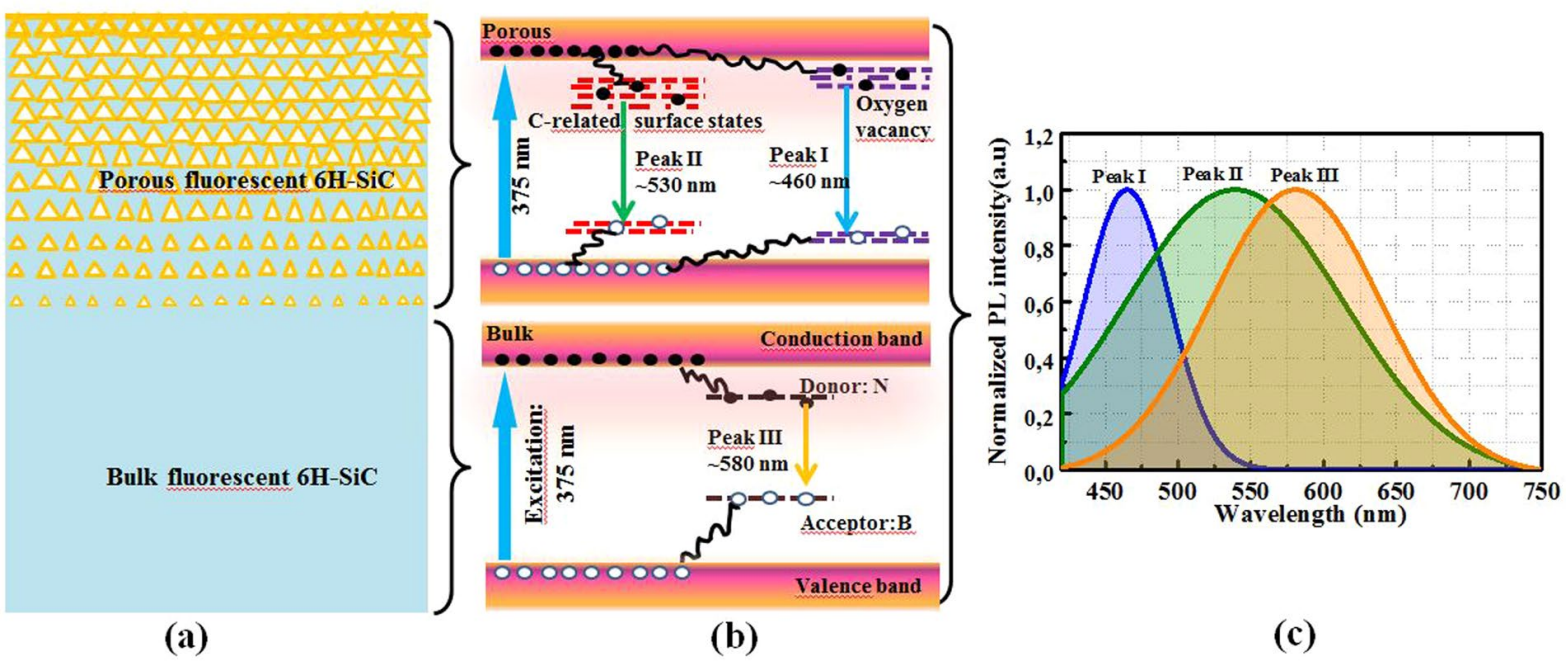
(**a**) Schematic diagram of the fluorescent 6H-SiC with porous surface layer. (**b**) Three possible transitions in fluorescent 6H-SiC: the DAP recombination (peak III) in bulk layer, the transition through C-related (peak II) and oxygen vacancy-related (peak I) surface defects in the porous layer. (**c**) Schematic of the normalized emission spectra from porous layer (peak I and peak II) and fluorescent bulk layer (peak III).

In brief, the carrier transitions in porous fluorescent 6H-SiC can be concluded with three radiative recombination channels, depicted in Fig. 7. The corresponding diagram of the porous surface layer on a fluorescent substrate is illustrated in Fig. 7(a). The three radiative recombination channels are attributed to the neutral oxygen vacancy defect state (peak I), non-bridging oxygen holes or C-related defects produced by the reaction of HF at the etched surface (peak II), and DAP recombination (peak III), respectively. For the porous layer, the radiative recombination is dominated by the defect states on the interface, i.e. peak I and peak II. As mentioned previously, the penetration depth of 375 nm in 6H-SiC is around 15 µm. When thickness of porous layer is thinner than that, the excitation light will generate peak I and peak II from porous layer, and peak III from the underneath fluorescent bulk layer. Hence, white light emission is realized by combining these three emission peaks.

## Conclusions

We have successfully achieved white light emission from fluorescent SiC that combines an N-B co-doped epitaxial layer with a porous surface layer. The room temperature photoluminescence spectra of the porous layer show two emission peaks centered approximately at 460 nm and 530 nm. Such blue-green emissions are attributed to neutral oxygen vacancies and interface Si/C related defects. The emission from the porous layer can be significantly enhanced by applying a thin ALD Al_2_O_3_ passivation layer. With a 10 µm thick porous layer, a high-quality white light was realized by combining the independent emissions of blue-green emission from the porous layer and yellow emission from the epitaxial N-B co-doped SiC layer. A CRI as high as 81.1 was achieved. In addition, porous fluorescent SiC offers a great flexibility in color rendering by tuning the thickness of porous layer on the surface and the underneath fluorescent bulk layer. Such novel approach opens a new perspective for the development of high performance white light emitting materials.

## Methods

### Anodic oxidation method

To achieve ohmic contacts on the backside of the SiC samples, layered nickel/titanium/gold films were prepared by sputtering or e-beam evaporation processes. Initially, the nickel layer with a thickness of 100 nm was deposited on the back side of the SiC samples via magnetron sputtering (CFs-4EP-LL, Shibaura Mechatronics Co., Japan), followed by a 4 min thermal annealing at 900 °C (Infrared Lamp Heating System RTA-4000). Then, 10 nm thick titanium and 200 nm thick gold films were evaporated on the top of nickel by an electron beam evaporation system (EI-5, ULVAC Co., USA). An anodic etching process was executed to form the porous layers on the surface of SiC substrate as described in our previous publication^26^. The samples were immersed in a 5% HF solution and exposed to UV irradiation of 365 nm by using mercury lamp. A constant pulsed current (1.25 mA, period: 50 ms, duty ratio: 50%) was injected between cathode (platinum counter electrode) and anode (metallic layers) for varied duration for different porous thickness. In order to improve the porosity uniformity, potassium persulfate (K_2_S_2_O_8_) was introduced during the fabrication of the porous fluorescent 6H-SiC samples with a concentration 0.015 mol/L.

### Atomic layer deposited Al_2_O_3_ films

In order to suppress the surface non-radiative recombination in the porous layer, an Al_2_O_3_ film was deposited on the pore surface by using ALD (Model R200, Picosun, Finland). The Al_2_O_3_ films were formed using H_2_O and trimethylaluminium (TMA:Al(CH_3_)) gases as precursors for the oxygen and aluminum, respectively. According to the optimized passivation condition for porous SiC^26^, 200 cycles were processed on the porous SiC samples, forming a 20 nm thick Al_2_O_3_ layer. Each ALD cycle contained 0.1 s pulse of TMA, 20 s N_2_ purge, 0.1 s H_2_O exposure followed by 20 s N_2_ purge. Post-deposition annealing was performed as 350 °C for 5 min in N_2_ ambient.

### Electron microscopy characterization

Cross-sectional images of the porous structures were acquired with a SEM (Zeiss Supra 40VP) microscope at 10 kV before passivation. Cross-sectional specimens were prepared perpendicular to the porous fabrication direction using the *in-situ* lift-out technique in a dual beam focussed ion beam (FIB)/SEM system (FEI Helios EBS3) equipped with a micromanipulator. A protective layer of platinum was deposited on the surface of the specimen, in order to protect the surface of porous layer from FIB-induced damage. Finally, the specimen surfaces were polished by a 2 kV Ga ion beam to minimize the damage caused by the 30 kV FIB. TEM images were acquired at 300 kV in a field-emission-gun TEM system (FEI Titan 80-300ST TEM), equipped with a solid state EDX detector.

### Measurements

The PL spectra were measured by a micro-PL setup. Both excitation light source (375 nm, power density 13.2 W/cm^2^) and detection optical spectrometer (CAS 140 B, Compact Array spectrometer, Instrument System) are fiber-coupled to an optical microscope (Olympus). The emission signal of the porous samples was filtered by a long-pass filter (cutoff wavelength 420 nm). Raman spectra were measured in a Raman spectrometer system (Horiba Jobin Yvon). The excitation laser wavelength is 532 nm with power of 10 mW, using a 100x objective. The signal collection time is 3 s and averaged 10 times.

### Data Availability

The datasets generated during and/or analysed during the current study are available from the corresponding author on reasonable request.
